# Interleukin-13 Signaling and Its Role in Asthma

**DOI:** 10.1097/WOX.0b013e31821188e0

**Published:** 2011-03-15

**Authors:** Efren L Rael, Richard F Lockey

**Affiliations:** 1 Department of Allergy, Asthma and Immunology, Penn State Hershey Medical Center MCH0401, Hershey, PA; 2 University Distinguished Health Professor, Professor of Medicine, Pediatrics & Public Health; Director, Division of Allergy & Immunology, Joy McCann Culverhouse Chair in Allergy & Immunology; World Allergy Organization President; University of South Florida College of Medicine University of South Florida; James A. Haley Veterans' Hospital, Department of A/I (111D), Tampa, FL

**Keywords:** interleukin-13, asthma, airway hyper-reactivity, fibrosis, single nucleotide polymorphism

## Abstract

Asthma affects nearly 300 million people worldwide. The majority respond to inhaled corticosteroid treatment with or without beta-adrenergic agonists. However, a subset of 5 to 10% with severe asthma do not respond optimally to these medications. Different phenotypes of asthma may explain why current therapies show limited benefits in subgroups of patients. Interleukin-13 is implicated as a central regulator in IgE synthesis, mucus hypersecretion, airway hyperresponsiveness, and fibrosis. Promising research suggests that the interleukin-13 pathway may be an important target in the treatment of the different asthma phenotypes.

## 

Asthma affects nearly 300 million people and one of every 250 deaths is attributed to this disease worldwide [1]. The cost of asthma hospitalizations, emergency room visits, lost school, and work days is significant. About 90% of patients respond to treatment regimens with inhaled corticosteroids with or without long-acting *β*2 agonists; how-ever, 5 to 10% seemingly do not. Treatment of this subgroup of patients accounts for greater than 50% of the total costs related to asthma [2,3].

Asthma is a reversible airway disease characterized by airway hyper-reactivity, inflammation and airway remodeling, and interleukin-13 (IL-13) is a recognized effector in these processes. For example, in a mouse model of asthma, IL-13 signaling results in mucin secretion, airway hyper-reactivity, fibrosis, and chitinase up-regulation [4].

Different phenotypes of asthma may explain why current therapies show limited benefits in subgroups of patients [5]. Patients with severe, poorly controlled asthma, who are insensitive to glucocorticoid treatment, highlight the need for new treatments. IL-13 signaling may be one pathway involved in the induction of corticosteroid-insensitive airway inflammation [6].

## IL-13 Structure and Signaling

IL-13 has a mass of 13 kDa and folds into 4 alpha helical bundles, A, B, C, and D. It shares overlapping secondary structural features with interleukin-4 (IL-4); however, it has 25% sequence homology and is capable of IL-4 independent signaling [7,8]. IL-13 signals through a shared receptor with IL-4 via a heterodimer receptor complex comprised of IL-4 receptor alpha (IL-4R*α*) and IL-13 receptor alpha 1 (IL-13R*α*1) (also termed the type 2 interleukin 4 receptor). Signaling through this receptor is initiated with high affinity when IL-13 binds IL-13R*α*1, leading to subsequent heterodimer formation with IL-4R*α*. Crystal structure analysis shows the heterodimer receptor complex signals with different potencies in response to IL-4 versus IL-13. This suggests that the extracellular cytokine-receptor interactions modulate intracellular membrane-proximal signaling events [9]. Both intracellular subunit receptor tails interact with tyrosine kinases of the Janus family (JAK 1-3, TYK2) [10]. IL-4R*α *associates with JAK1 whereas IL-13R*α*1 interacts with either JAK2 or TYK2, but not JAK3. Once JAK1 is activated, the IL-4R*α *tyrosine residues in the cytoplasmic domain are phosphorylated enabling the transcription factor STAT6 to dock. Once phosphorylated, 2 STAT6 molecules dimerize and translocate to the nucleus where the complex affects transcription of many IL-13 dependent genes.

**Figure 1:**
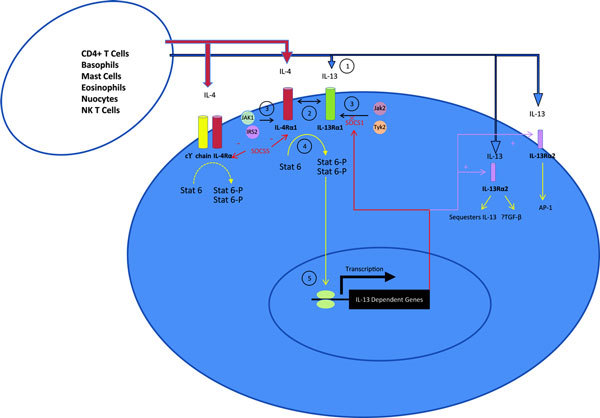
**IL-13 signaling**. Step 1, the binding of IL-13 to IL-13R*α*1 leads to step 2, heterodimer formation with IL-4R*α*1 and formation of the type 2 IL-4 receptor. Step 3 leads to Janus Kinase activation (JAK), followed by step 4, STAT6 phosphorylation, dimerization, and translocation to the nucleus. In step 5, Stat 6 heterodimers affect IL-13-dependent gene transcription. IL-13R*α*2 is an IL-13 dependent gene. IL-13 can bind IL-13R*α*2 that leads to sequestration of IL-13 or IL-13 signaling by TGF-*β *induction or AP-1 signaling. Mouse models suggest that suppressor of cytokine signaling (SOCS)1 is an IL-13 dependent gene that interacts with JAK2 to negatively regulate JAK2 association with IL-13R*α*1 [14]. In a mouse model, SOCS5 targets IL-4R*α*1 and impairs STAT6 signaling [15].

IL-13 can also bind a high affinity receptor, IL-13 receptor alpha 2 (IL-13R*α*2). In the mouse, this receptor has a soluble form and a membrane bound form resulting from alternative transcriptional splice variants. IL-13R*α*2 in humans is primarily an intracellular rather than a membrane-bound molecule in both primary bronchial epithelial cells and fibroblasts and displays a diffuse granular cytoplasmic distribution in both cell types [11]. It is thought to act as a decoy receptor because of its short cytoplasmic tail. However, studies suggest that this receptor mediates IL-13 signaling and induces TGF-*β *production in both in vitro human and mouse and in vivo mouse experiments (Figure 1) [12,13].

Extracellular regions of the IL-13R*α*1 and 2 domains in mice are composed of 3 fibronectin type III domains D1, D2, and D3. The D2 and D3 domains have cytokine receptor homology modules (CRHs), which show similar structure to the class I cytokine receptor superfamily. Based on mutation studies, the IL-13R*α*1 CRH module locations at leucine 319 and tyrosine 321 are important for binding to IL-13. So too, the IL-13R*α*2 CRH module locations at tyrosine 207, aspartate 271, tyrosine 315, and aspartate 318 are important for binding to IL-13 [16].

Through DNA mutagenesis studies, it was shown that the alpha helices in IL-13 A, C, and D participate in interaction with IL-13R*α*1 receptors [17,18]. Lysine residues 105, 106, and arginine 109 of the D-helix of IL-13 interact with IL-13R*α*2. Glutamic acids at position 92 and 110 and leucine at position 104 are important for IL-13/IL-4 receptor stimulation [18].

IL-13 is produced by CD4^+ ^T cells, NK T cells, mast cells, basophils, eosinophils, and nuocytes [17,21]. It is implicated as a central regulator in IgE synthesis, mucus hypersecretion, airway hyperresponsiveness (AHR), and fibrosis [4].

Through the use of the knockout mouse model, airway resistance, mucus production, and profibrogenic mediator induction are nearly totally dependent on IL-13R*α*1, which serves as a signaling molecule for both IL-4 and IL-13 [4]. Allergen-induced TGF-*β *is completely dependent on IL-13R*α*1.

## IL-13 Role in Mucus Production

Goblet cell hyperplasia and mucus overproduction are features of asthma and chronic obstructive pulmonary disease and can lead to airway plugging, a pathologic feature of fatal asthma [21-23]. Animal models demonstrate that IL-13 induces goblet cell hyperplasia and mucus hypersecretion [24,25].

Human bronchial epithelial cells (HBEs), stimulated by IL-4 and IL-13, can also undergo changes from a fluid absorptive state to a hypersecretory state independent of goblet cell density changes [26,27].

Human in vitro studies demonstrate that IL-13 induces goblet cell hyperplasia, increases bronchial epithelial periodic acid schiff (PAS) cell staining, and MUC5AC expression. Experiments suggest that these effects are mediated by IL-13 signaling through IL-13R*α*1 [28,29]. In these experiments, IL-13 also led to an increase in the soluble form of IL-13R*α*2. IL-13R*α*2 decreased the quantity of PAS+ cells, MUC5AC+ cells, goblet cells, and decreased both the mRNA expression and protein secretion of MUC5AC induced by IL-13 [29].

Antibody blockade of IL-13R*α*2, in the presence of IL-13, on HBEs, led to increased PAS+ epithelial cells, goblet cells and MUC5AC+ cells and MUC5AC mRNA expression. This suggests that the soluble form of IL-13R*α*2 may negatively modulate IL-13 signaling [29]. IL-4 was also able to increase the number of PAS+ cells, goblet cells and MUC5AC+ cells; however, IL-13R*α*2 had no effects on the number of PAS+ cells, goblet cells and MUC5AC+ cells, MUC5AC mRNA expression or protein secretion induced by IL-4 [29].

In human airway epithelial cells, IL-13 is known to induce 15-Lipoxygenase-1 (15-LO1), an important enzyme in the arachidonic acid pathway, that forms stable 15-hydroxy-eicosatetraenoic acid (15-HETE) from metabolism of arachidonic acid. Human epithelial 15-LO1 expression is correlated with asthma severity [30]. IL-13 induction of 15-LO1 stimulates formation of 15-HETE that can be further metabolized through esterification to phosphatidylethanolamine (15-HETE-PE). IL-13 induction of 15-HETE-PE enhances MUC5AC expression in human airway epithelial cells [30].

## IL-13 Role in Aspirin Exacerbated Respiratory Disease (AERD)

The triad of aspirin sensitivity, asthma, and nasal polyposis has been documented since at least 1922 [31]. About 10% of adults with asthma will experience a flare of their asthma accompanied by naso-ocular reactions after ingestion of aspirin or nonsteroidal anti-inflammatory drugs (NSAIDS) leading to intense eosinophilic inflammation of the nasobronchial tissues and cysteinyl-leukotriene (Cys-LTs) overproduction [33]. Polymorphisms within the *IL-13 *gene are associated with increased eotaxin-1 levels, increased eosinophil count, and the development of rhinosinusitis in patients with AERD [33].

**Figure 2:**
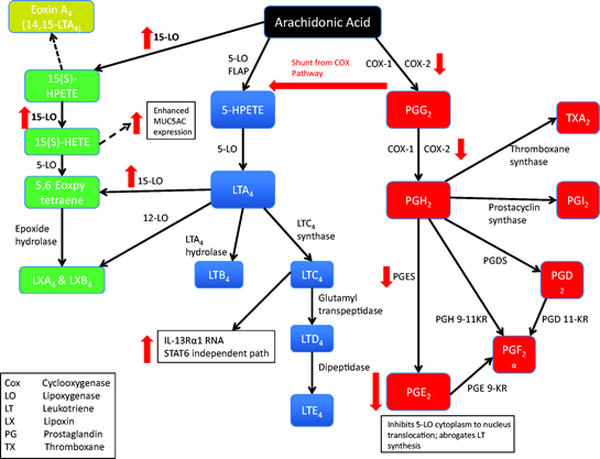
**Arachidonic acid metabolic pathways and the role of IL-13**. The prostaglandin pathway (red), the leukotriene pathway (blue), and the lipoxin pathway (green). PGE2 has inhibitory effects on leukotriene production including inhibition of 5-LO translocation from the cytoplasm to the nucleus and abrogation of leukotriene synthesis [24]. IL-13 down-regulates PGE2 both directly and indirectly through effects on PGES and COX-2. IL-13 also induces 15-LO that is correlated with asthma severity. IL-13 induction of 15-LO stimulates formation of 15-HETE and a metabolite of this enhances MUC5AC expression in human airway epithelial cells.

IL-13 is implicated in regulating key arachidonic acid metabolic pathways including the prostaglandin pathway, the leukotriene pathway, and the lipoxin pathway (Figure 2).

Interestingly, IL-4 increased LTC4 Synthase expression, a protein important in converting LTA4 to LTC4 that is most implicated in AERD; whereas IL-13 had no effect on LTC4 Synthase up-regulation [35]. LTC4 increased IL-13R*α*1 RNA levels via a STAT6 independent pathway [36]. IL-13 appeared to have both upstream and downstream effects. In a mouse model, intranasal IL-13 induced an increase in Cys-LT receptors 1 and 2 RNA and decreased Cys-LT production at a dose of 0.035 *μ*g [36]. At higher doses, intratracheal IL-13 induced an increase in 5-Lipoxygenase (5-LO) mRNA and increased BALF Cys-LTs (LTC4, D4, E4) within 15 minutes and LTB4 within 6 hours in BP2 mice [37].

IL-13 inhibits mPGES-1 (prostaglandin E synthase membrane-bound form) and COX-2 and up-regulates 15-prostaglandin-dehydrogenase (15-PGDH), the PGE-2 metabolizing enzyme, thereby leading to decreased PGE-2 levels [38]. PGE-2 regulates wound closure in airway epithelium, and exogenous application of PGE-2 stimulates wound closure, a vital process in airway epithelial repair after injury, maintenance of barrier function and limitation of airway hyperreactivity [39].

Lipoxins are important in inflammation resolution, acting locally at sites of inflammation. Administration of intrapleural lipoxin A4 (LXA4) to OVA sensitized rats, decreased allergen-induced pleural eosinophil and neutrophil infiltration [40]. In vitro treatment of the GM-CSF stimulated eosinophilic leukemia cell line (EoL-1) cells with LXA4 inhibited secretion of IL-8, IL-13, and eotaxin suggesting this molecule may have negative feedback implications on IL-13 mediated pathways [41].

## Interleukin-13 in Pulmonary Fibrosis

Fibrosis is a major cause of morbidity and mortality with limited treatment options. Chronic inflammation induced by allergens, infections, toxins, autoimmune reactions, radiation, and mechanical injury can result in architectural lung changes, gas exchange impairment, and fibrosis with heterogeneous temporal development depending on the trigger. Experimental models identify IL-13 to be an important profibrotic mediator [25,42-50].

Transgenic mouse in vivo studies demonstrate that lung over-expression of IL-13 induces subepithelial airway fibrosis without additional stimulus [25]. Antibody blockade of IL-13 in mouse lungs challenged with *Aspergillus fumigatus conidia*, to simulate chronic allergic asthma, or bleomycin, to simulate toxin mediated fibrosis, led to decreased lung collagen deposition [42,43].

IL-13 induces CC-chemokines, including CCL3 (MIP-1*α*), CCL4 (MIP-1*β*), CCL20 (MIP-3 *α*), CCL2 (MCP-1), CCL11(eotaxin), CCL22 (MDC), and CCL6 (C10) [43,44]. Antibodies to CCL3, CCL2, and CCL6 profoundly abrogate lung remodeling responses in IL-13 transgenic mice and in bleomycin challenged mice, suggesting the pathogenesis of fibrosis has nonredundant mechanisms [43,44].

Both IL-4 and IL-13 have redundant signaling pathways with implications in pulmonary fibrosis. IL-4 and IL-13 are important in alternatively activated macrophage induction, which is believed to regulate fibrosis [45]. The use of knock-out mice deficient in IL-13R-1 implicates this receptor as the most important fibrosis signaling mechanism in fibrosis development [46]. Furthermore, several experimental models of fibrosis suggest that IL-13 is the dominant effector in toxin, [47,48] infection, [49] allergic, [42,50] and posttransplant bronchiolitis obliterans [51] models of fibrosis.

In addition to the redundancy of IL-4 and IL-13 signaling through IL-4R*α*/Stat6, IL-13 may signal through a distinct pathway via the IL-13R*α*2 driving TGF*β*1 dependent pulmonary fibrosis [13]. IL-13 induces latent TGF*β*1 production in macrophages. TGF*β*1 forms homodimers that are noncovalently bound to a latency-associated protein (LAP) when stored in an inactive form in cells. IL-13 also indirectly activates TGF*β *by up-regulating LAP cleavage proteins [52,53]. Additionally, IL-13 activates TGF*β *through stimulation of matrix metalloproteinases and cathepsin-based proteolytic pathways [53,54]. In IL-13 transgenic mice, TGF*β*1 plays a key role in subepithelial fibrosis evolution [52].

## IL-13 in Airway Hyperresponsiveness

CD4^+ ^T lymphocytes are important in the events leading to AHR in animal asthma models, via an IL-4R, STAT6 mechanism [55,56]. This process is independent of IL-4 and IL-5. Exogenous addition of IL-13 in T lymphocyte-deficient mice promotes AHR and airway inflammation [57].

IL-13 modulates Ca2+ responses in vitro in human airway smooth muscle through a STAT6/JAK-independent mechanism [58]. It signals through MAP kinases ERK and JNK effecting airway smooth muscle, leading some to hypothesize that STAT6 dependent pathways may be important in acute AHR, whereas STAT6-independent AHR and airway remodeling mechanisms may be important in chronic models [58-60].

Inflammation, remodeling, and AHR, all features of asthma, are induced by IL-13 overexpression [24,25]. Blockade of IL-13 by the soluble receptor-Fc fusion protein abrogates allergen-induced AHR [24,56].

IL-13 is overexpressed in sputum, bronchial submucosa, peripheral blood, and mast cells in the airway smooth muscle bundle in asthmatics further supporting its role in the pathogenesis of AHR [61-64]. Human IL-13 mRNA elevation in bronchial biopsy homogenates is seen in both atopic and nonatopic asthmatics versus nonasthmatics in RT-PCR experiments [65]. In situ hybridization studies of bronchial biopsy specimens from steroid responsive asthmatics versus steroid resistant asthmatics treated with one week of prednisolone demonstrated a decrease in IL-13 mRNA+ cells after treatment in the steroid responsive group that correlated with asthma clinical improvement in contrast to the steroid resistant group that maintained IL-13 mRNA+ cells and no clinical response [66]. Sputum IL-13 levels correspond to airway eosinophil percentage that is associated with airway inflammation in corticosteroid-naive subjects [67]. Furthermore, allergen challenged mild asthmatics have up-regulated IL-13 concentrations in bronchoalveolar lavage [68].

Sputum IL-13 concentration and the number of IL-13+ cells in the bronchial submucosa and airway smooth muscle bundle is increased in severe asthmatics [69]. Additionally, sputum IL-13 concentration is negatively associated with asthma control [69]. Steroid-responsive asthmatics, treated with oral steroids for 1 week, demonstrate decreased bronchial biopsy specimen IL-13 mRNA expression. However, patients clinically steroid nonresponsive, demonstrate persistent IL-13 mRNA expression [65]. This observation of persistent IL-13 sputum levels and bronchial biopsy specimen levels was observed in 2 cohorts of patients treated with intramuscular triamcinolone, to exclude noncompliance as a confounding variable [69].

## IL-13 in Glucocorticoid Resistant Asthma

Glucocorticoids are anti-inflammatory medications of-ten used as maintenance therapy in acute and chronic asthma; however, some patients with severe asthma are steroid non-responsive. IL-13 remains elevated in glucocorticoid (GC) insensitive asthma, but not GC sensitive asthma [70,71].

GCs enter the cell by diffusion across the cell membrane with subsequent binding to the glucocorticoid receptor (GR), which dimerizes in the cytoplasm. The complex then translocates to the nucleus by nuclear transport proteins, whereby the complex modulates transcription of many genes pertaining to asthma through both activation and repression.

GCs have been shown to repress IL-13 transcription indirectly. In vitro experiments demonstrate that fluticasone can inhibit the IL-13 activating transcription factor GATA-3, from translocating into the nucleus through competition for the nuclear translocation protein importin *α*, in the T lymphocyte cell line HuT-78 and in peripheral blood mononuclear cells (PBMC) [72]. Additionally, fluticasone can induce expression of mitogen-activated protein kinase (MAPK) inhibitor phosphatase-1 (MKP-1), an inhibitor of p38 MAPK which is required for phosphorylation of GATA-3 before GATA-3 binding to importin *α *and transport into the cell nucleus [72].

GCs can also repress IL-13 transcription directly within the nucleus. The GC receptor has 2 isoforms designated GR*α *and GR*β *based on alternative splice sites [73]. Both GR*α *and GR*β *repress transcription of IL-13 [74]. GCs repress IL-13 gene transcription within the cell nucleus, in part, by competitively inhibiting activation mediated by NF-AT/AP-1 DNA binding sites in the proximal promoter [75]. GR*β *mediates repressive function through the recruitment of histone deacetylase com-plexes [74]. Interestingly, higher numbers of GR*β *immunoreactive cells in the bronchoalveolar lavage fluid and peripheral blood were identified in patients with GC-insensitive asthma [71]. Given that elevated levels of IL-13 are identified in GC-insensitive asthmatics, GR*β *inability to down-regulate IL-13 may be responsible for this finding; however, no known studies have been performed to answer this question.

GC effects can be regulated at many steps within the cell. IL-2, IL-4, and IL-13 have all been found to be up-regulated in patients with steroid-resistant asthma [66,68,76]. Upon cell entry, endogenous GCs can be converted to an active or inactive form by the enzyme 11*β*-hydroxysteroid dehydrogenase (11*β*-HSD)-1 and 11*β*-HSD-2, respectively, thereby regulating the bioavailability of the GC substrate for the GR. IL-13 can up-regulate 11*β*-HSD-1 and this is thought to act as a negative feedback loop to curtail inflammation through the steroid anti-inflammatory effects previously mentioned [77]. It is unknown whether this pathway plays a role in steroid-resistant asthma.

In vitro, GC receptor function and binding affinity for GCs are theorized to be reduced by GR phosphorylation events within the cytoplasm. One such kinase thought to be important in GR phosphorylation is p38 MAPK, which is activated by IL-13, IL-2, and IL-4, since p38 MAPK inhibitor abrogates these effects [78].

## IL-13 in Chitin Allergic Asthma

Chitin, the second most abundant biopolymer in nature, consists of N-acetyl-ß-D-glucosamine, and provides structural rigidity to fungi, crustaceans, helminthes, and insects [79]. Many chitin containing organisms have been implicated in allergy and asthma. Characterization of dust mite and cockroach anatomy, demonstrates chitin makes up a large component of the exoskeleton. Chitin can be introduced into the airway by inhalation of exoskeletons of dust mites, cockroaches, and by inhalation of fungi like *Aspergillus *and Alternaria.

Humans do not synthesize chitin, however, the human genome encodes for chitinases (enzymes that cleave chitin), and chitinase-like proteins (proteins that bind but do not cleave chitin) [80]. These proteins are hypothesized to counter parasitic infection [80]. The chitinase and chitinase-like proteins (C/CLP) are referred to as the 18-glycosyl hydrolase family [80]. In humans, the chitinase and chitinase-like proteins include Acid Mammalian Chitinase (AMCase), chitotriosidase, oviductin, HcGP-39/YKL-40 (chitinase 3-like-1), and YKL-39 (chitinase 3-like-2) [80].

In the mouse model, chitin challenge can induce accumulation of IL-4 expressing innate immune cells including eosinophils and basophils to the site of challenge, to the lungs or intraperitoneally, in a tissue nonspecific manner [81]. Mouse models of bronchial asthma demonstrate that AMCase is involved in the pathophysiology of asthma. AMCase has been shown to be induced by a T Helper-2 (T_H_2)-specific, IL-13-mediated pathway in epithelial cells and macrophages after ovalbumin sensitization and aerosol lung challenge in mice [82]. Additionally, AMCase mRNA was highly induced after *A. fumigatus *intranasal challenge in mice [83]. Inhibition of AMCase leads to abrogated T_H_2 inflammation, less bronchial hyper reactivity, and fewer eosinophils [84].

Through a series of experiments with knockout models of IL-13 signaling, including knockout mice lacking IL-13R*α*1 or IL-4R*α*, Munitz et al, demonstrated that key pathogenic molecules associated with asthma severity, such as chitinase, are entirely dependent on IL-13 signaling through IL-13R*α*1, a component of the type II receptor.

In human lung autopsies, AMCase is expressed in exaggerated quantities in asthmatic lung epithelial cells and macrophages versus nonasthmatic lungs [82]. Interestingly, Siebold et al, reports both lower AMCase protein levels in BAL fluid and lower AMCase protein activity from asthmatic subjects with mild-to-moderate asthma compared with normal subjects suggesting a protective role of functional AMCase [85].

Investigation by a group at Yale, showed that asthma severity can be correlated with YKL-40 (chitinase 3-like-1) levels [86]. YKL-40 has been shown to be up-regulated by IL-13 [87].

## IL-13 in Infectious Asthma

Infections are implicated in asthma flares [5]. Rhinovirus (RV), the virus responsible for the common cold, in children is a distinct risk factor for asthma exacerbations with an odds ratio of 6.8 [88]. A Finnish study compared cytokine responses in acute and convalescent rhinovirus versus respiratory syncytial virus (RSV) induced early wheezing in hospitalized, steroid-naive patients aged 3-35 months. Among the cytokines studied, IL-13 was the most increased in the RV group versus the RSV group with a 39-fold increase in the acute phase and 33-fold increase in the convalescent phase [89].

IL-13 is implicated in innate immune responses independent of IgE or T lymphocytes. In vitro, human mast cell activation with thymic stromal lymphopoietin (TSLP) or Toll-Like Receptor (TLR) 2 activated airway epithelial cells, in addition to IL-1, led to IL-5 and IL-13 production [90]. In mice, bone marrow derived mast cells stimulated with *Staphylococcus aureus *derived peptidoglycan, in a TLR2-dependent process, produced IL-13, IL-4, IL-5, IL-6, and TNF-*α*. Bone marrow derived mast cells stimulated with lipopolysaccharide derived from *Escherichia coli*, in a TLR4-dependent process, produced IL-13, TNF-*α*, IL-1*β *, and IL-6, but not IL-4 nor IL-5 [91]. In vitro HBE cells exposed to IL-4 and IL-13, mount decreased cellular antimicrobial activity and decreased mRNA levels of the antimicrobial human *β*-defensin 2 but not *β*-defensin 1 or LL-37 [92].

## IL-13 Signaling Polymorphisms and Asthma

More than 10 papers report an association between single nucleotide polymorphisms (SNP) in IL-13 and the effects on asthma in adults and in children, in the context of infections, atopy, IgE levels, or risk for asthma (Table 1) [93-103]. Studies utilizing haplotype and multigene analysis have made similar associations of IL-13 SNPs and asthma (Table 2) [95,100,104].

**Table 1:** IL-13 Single-Nucleotide Polymorphisms (SNP) Associated With Asthma

Gene	SNP(s)/SNP ID#	SNP Associated Phenotype	Race Background	*P *Value if Available	Reference
IL13	Arg130Gln	Atopy	Multi-center	0.01	([93])
		Total eosinophil, total serum IgE level	Childhood asthmatics with mild to moderate asthma	0.0442	
	Arg130Gln (= G4257A)	Atopic dermatitis	Japanese aged 11-61	0.043	([94])
	Arg130Gln/IL4C-589T	Atopy	Canadian children with family	0.006	([95])
	Haplotype		history of asthma		
	Arg130Gln	Atopy	Iceland chart review	0.67	([96])
	Arg130Gln	Late wheeze age 6	Dutch children	0.007	([97])
	Arg130Gln	Asthma	British young	0.017 B	([98])
		No association with IgE levels	Japanese young adults	0.026 J	
	Arg130Gln	Total serum IgE	German children	0.005	([99])
	- 1112C/T			0.0002	
	- 1112C/T rs1800925	Severe RSV infection asthma	German children < 2 hospitalized for RSV	0.026	([100])
	- 1112C/T	Asthma	Dutch	.005 asthma	([101])
		BHR		.003 BHR	
		+ Skin test		.03 + ST	
	- 646A/G	FEV1 postbronchodilator	African American	0.009	([102])
IL4R*α*	Gln551Arg	Atopic asthma	Meta analysis combo adult + children	OR 1.6; *P *= 0.004	([103])

BHR = Bronchial hyper-reactivity; B = British cohort; J = Japanese cohort.

**Table 2:** Haplotype/Multi-Gene Analysis With Inclusion of the Arg130Gln IL-13 SNP and the Associated Haplotype Phenotype

Gene	SNPs	rs Signatures	Haplotype Associated Phenotype	Race Background	*P *Value if Available	Reference
IL13	- 1512A/C	rs1881457	Severe RSV infection asthma	German children < 2 years old,	0.009 (FASTEHPLUS)	([100])
	- 1112C/T	rs1800925		hospitalized for RSV	0.01183 (FAMHAP)	
	Arg130Gln	rs20541				
IL4 + IL13	- 589C/T	rs2243250	Severe RSV infection asthma	German children < 2 years old,	0.0008 (FASTEHPLUS)	([100])
	- 1512A/C	rs1881457		hospitalized for RSV	0.0011 (FAMHAP)	
	- 1112C/T	rs1800925				
	Arg130Gln	rs20541				
IL4R*α *+ IL13	Ile50Val Arg130Gln		Asthma + IgE levels	Chinese children 5 to 18 years of age	0.013 (GMDR)	([104])
IL4 + IL13	- 589C/T		Atopic dermatitis	Canadian children with family	0.006	([95])
	Arg130Gln		Atopy (Asthma not investigated)	history of asthma or atopy	0.009	

GMDR = Generalized multifactor dimensionality reduction method.

Polymorphisms in the *IL-13 *gene associated with asthma are described at Arg130Gln (rs20541) (also described as Arg164Gln, Gln110Arg, +2044 NIaIV RFLP, and Arg144Gln based on IPI, Unit-ProtKB/Swiss-Prot, EMBL CDS databases) in a coding region of the *IL-13 *gene [19,97,105]. This leads to substitution of a positively charged arginine with a neutral glutamine and is the only nonsynonymous substitution present in all ethnic groups thus far studied (East and West Africa, Europe, China, and South America). Studies suggest that the Arg130Gln substitution results in decreased affinity of IL-13 for IL-13R*α*2, increased expression of IL-13 and phosphorylation of STAT6 [16,106]. In a predominantly White cohort of 9960 people from Washington County, MD, the GG genotype frequency was 64%, the GA genotype frequency was 29%, the AA genotype frequency was 4%, with 3% samples missing. Frequency percentages were equivalent between male and female genders [107]. Furthermore, IL-13 Arg130Gln SNP did not show variation in allele frequency by race [108].

Polymorphisms are present in IL-13 and in the cognate receptors. A longitudinal Dutch family study population with asthma, followed an average of 26.5 years, with polymorphisms in both the *IL-4Rα *gene (S478P) and *IL-13 *at the-1112 promoter region (also described as - 1111,-1055) (rs1800925) demonstrate an odds ratio 4.87 times greater for the development of asthma versus individuals without these associated genotypes (*P *= 0.0004) [109]. The association of the C-1112T promoter polymorphism with asthma is reported in diverse ethnic populations with the allergic phenotype. Functional analysis of the promoter SNP identified a Yin-Yang 1 binding site activator that overlapped with a STAT motif repressor. The Yin-Yang 1 binding site is hypothesized to increase IL-13 transcription as opposed to STAT6 mediated repression of IL-13 transcription in T_H_2 cells [110]. However, these results did not carry over to nonpolarized CD4^+ ^T cells. The type 2 interleukin 4 receptor is dose dependent in response patterns to IL-13 and IL-4 and transcriptional dysregulation at the promoter could have profound effects on IL-13 signaling.

A single nucleotide polymorphism in the IL-13 cognate receptor, IL4R*α*, based on meta-analysis, identified the Q551R (+1652 A/G, rs1801275) IL4R variant to impart a combined OR, 1.6; *P *= 0.004 for risk of atopic asthma. The amino acid residue 55 is located in the cytoplasmic domain in the vicinity of the STAT6 binding site and is hypothesized to affect STAT6 signal transduction.

## Clinical Trials Targeting IL-13/IL-4 Signaling

Clinical trials are currently underway to investigate blockade of IL-13/IL-13 signaling in the treatment of asthma (Table 3). A phase IIa clinical trial of a recombinant human IL-4 variant, pitrakinra AER 001 (AEROVANT, by Aerovance, Berkeley, CA) that competitively binds the IL-4R*α *complex and interferes with IL-4 and IL-13 actions was published in 2007 [111]. The results demonstrate a reduction in the late phase response, after allergen challenge in allergic asthmatics, measured as a decreased fall in forced expiratory volume in 1 second (FEV1) and the area under the FEV1 time curve.

**Table 3:** Clinical Trials Targeting IL-13/IL-4 Signaling

Agent	Company	Mechanism	Status
CAT-354	Cambridge Antibody Technology, Medimmune/AstraZeneca	Anti-IL-13 mAb	Phase IIa
QAX576	Novartis	Anti-IL-13 mAb	Completed phase II
IMA-638 (Anrukinzumab)	Wyeth	Humanized Anti-IL-13 mAb	Completed phase II
IMA-026	Wyeth	Anti-IL-13 mAb	Phase II
MILR1444A (Lebrikizumab)	Genentech	Humanized Anti-IL-13 mAb	Phase II
AIR645	Altair Therapeutics	Anti-sense targeting mRNA for IL4R*α*1	Phase IIa
AER001 (Pitrakinra)	Aerovance	Human IL-4 mutein inhibits IL13R*α*1 or IL2R*γ*) from assembly into receptor complexes w/IL4R*α*	Completed phase II
AMG317	Amgen	Human Anti-IL-4R*α *mAb	Completed phase II

mAb = monoclonal antibody.

Anti-IL-13 monoclonal antibody CAT-354 (NCT00873860, by Cambridge Antibody Technology, 2006 acquired by Astra Zeneca, 2007 merged with MedImmune, Gaithersburg, MD) is in phase IIa investigation to study the efficacy and safety in adults with uncontrolled moderate-to-severe, persistent asthma.

Anti-IL-13 monoclonal antibody QAX576 (NCT00532233, by Novartis, Basel Switzerland) completed phase II clinical trials recruiting patients with idiopathic pulmonary fibrosis to treatment with single dose QAX576 to measure IL-13 production.

A 2'O-methoxyethyl second-generation antisense drug targeting IL4R*α*1 mRNA AIR645 (NCT00941577, by Altair Therapeutics, Inc., San Diego, CA) is recruiting subjects with mild allergic asthma and is in phase IIa investigation.

A humanized mouse monoclonal antibody against human IL-13, IMA-638 (Wyeth, 2009 acquired by Pfizer, New York City, NY), completed 2 phase II investigations in subjects with persistent asthma (NCT00425061) and in subjects with mild atopic asthma (NCT00410280). Wyeth has another monoclonal antibody against human IL-13, IMA-026 that is in phase II clinical trials after completion of phase I investigation on allergen-induced late asthma responses in subjects with mild asthma (NCT00725582).

A humanized monoclonal antibody against IL-13, Lebrikizumab (NCT00930163, by Genentech, 2009 acquired by F. Hoffmann-La Roche Ltd., Basel, Switzerland), is in phase II investigation in asthmatic adults inadequately controlled with inhaled corticosteroids.

A human monoclonal antibody against IL4R*α*, AMG317 (NCT00436670, by Amgen, Thousand Oaks, CA) has completed phase II investigation on safety and efficacy in subjects with moderate to severe asthma.

## Caveats in ANTI-IL-13 Therapeutics

CD4^+ ^T_H_17 cells produce the cytokines IL-17A and IL-21, which when dysregulated, are implicated in inflammatory and autoimmune diseases. In a mouse model, IL-13 negatively regulates IL-17A and IL-21 production by downregulating expression of the transcription factor retinoic acidrelated *γ*T (ROR-*γ*t) [112]. Newcomb DC et al, speculate that blockade of IL-13 signaling could result in up-regulation of T_H_17 inflammation in disease states associated with altered T_H_17 signaling [112]. Evidence to support this hypothesis is demonstrated in double knock-out IL-4/IL-13 mice epicutaneously sensitized with OVA that resulted in increased systemic T_H_17 responses that affected the lungs after antigen challenge, and resulted in airway inflammation and airway hyperresponsiveness [113].

Further work is needed to define asthma phenotypes. Woodruff PG et al, reports IL-13 blockade may benefit only a subset of asthmatics [114]. In this asthma cohort, half of the asthmatics had IL-13 induced epithelial gene expression profiles in vitro and responses to inhaled corticosteroids, while the other half did not respond despite similar clinical phenotypes and the presence of atopy. Woodruff PG et al, speculate that airway barrier function defects, IL-17 induced inflammation, neutrophilic inflammation, and infection are important mechanisms that warrant further investigation.

## Conclusions

IL-13 is an important cytokine in airway hyperresponsiveness, mucus production, airway remodeling, subepithelial airway fibrosis, infectious asthma, allergic asthma, and aspirin exacerbated respiratory disease. Research studies have implicated this cytokine in many of the pathologic events in different asthma phenotypes. As with many biologic events, often there are countervailing regulatory signals for activation and inactivation. It will be important to further understand both the mechanisms by which IL-13 is activated and inactivated and how these signaling pathways play into the bigger picture of cytokine networks, gene-gene interactions, and ultimate pathology.

### Search Strategy and Selection Criteria

This Seminar is based on PubMed, ISI Web of Knowledge search for articles with "interleukin-13" in conjunction with "asthma," "airway hyperreactivity," "fibrosis," "single nucleotide polymorphism," "chitin," "aspirin exacerbated respiratory disease," "aspirin," "signaling," "mucus," glucocorticoid," "infection" in combination with "treatment" keywords, and other review articles and references from those review articles deemed relevant. Clinical trials were identified by using the keyword "interleukin-13" in clinicaltrials.gov. We prioritized more recent publications; however, there was no restriction on language or date of publication.

## Notes

1. These amino acids are also enumerated as Lysine 137, 138; Arginine 141, Glutamic acids 124 and 142 in the International Protein Index (IPI), UniProtKB/Swiss-Prot, EMBL CDS protein sequence repositories [19].

## End Note

Contributors: ELR contributed to design, content, literature review, and writing. RFL contributed to structure, editing, and content.
